# Studying the Drug Delivery Kinetics of a Nanoporous Matrix Using a MIP-Based Thermal Sensing Platform

**DOI:** 10.3390/polym9110560

**Published:** 2017-10-28

**Authors:** Christopher J. Pawley, Ariane Perez-Gavilan, Kaelin S. Foley, Sarah Lentink, Hannah N. Welsh, Gabrielle Tuijthof, Erik Steen Redeker, Hanne Diliën, Kasper Eersels, Bart van Grinsven, Thomas J. Cleij

**Affiliations:** 1 Maastricht Science Programme, Maastricht University, P.O. Box 616, 6200 MD Maastricht, The Netherlands; c14354791@mydit.ie (K.S.F.); s.lentink@alumni.maastrichtuniversity.nl (S.L.); h.welsh@alumni.maastrichtuniversity.nl (H.N.W.); erik.steenredeker@maastrichtuniversity.nl (E.S.R.); hanne.dilien@maastrichtuniversity.nl (H.D.); kasper.eersels@maastrichtuniversity.nl (K.E.); bart.vangrinsven@maastrichtuniversity.nl (B.v.G.); thomas.cleij@maastrichtuniversity.nl (T.J.C.); 2 Dublin Institute of Technology, School of Chemical and Pharmaceutical Sciences, Kevin Street, D08 X622 Dublin 2, Ireland; 453477@dit.ie; 3 Zuyd University of Applied Science, Faculty of Beta Sciences and Technology, Smart devices unit, Nieuw Eyckholt 300, 6419 DJ Heerlen, The Netherlands; gabrielle.tuijthof@zuyd.nl

**Keywords:** molecularly imprinted polymers, thermal detection, nanoporous matrix, drug delivery

## Abstract

The implementation of Molecularly Imprinted Polymers (MIPs) into sensing systems has been demonstrated abundantly over the past few decades. In this article, a novel application for an MIP-based thermal sensing platform is introduced by using the sensor to characterize the drug release kinetics of a nanoporous silver-organic framework. This Ag nanoporous matrix was loaded with acetylsalicylic acid (aspirin) which was used as a model drug compound in this study. The drug elution properties were studied by placing the nanoporous matrix in phosphate buffered saline solution for two days and measuring the drug concentration at regular time intervals. To this extent, an acrylamide-based MIP was synthesized that was able to detect aspirin in a specific and selective manner. Rebinding of the template to the MIP was analyzed using a thermal sensor platform. The results illustrate that the addition of aspirin into the sensing chamber leads to a concentration-dependent increase in the phase shift of a thermal wave that propagates through the MIP-coated sensor chip. After constructing a dose-response curve, this system was used to study the drug release kinetics of the nanoporous matrix, clearly demonstrating that the metalorganic framework releases the drug steadily over the course of the first hour, after which the concentration reaches a plateau. These findings were further confirmed by UV–Visible spectroscopy, illustrating a similar time-dependent release in the same concentration range, which demonstrates that the MIP-based platform can indeed be used as a low-cost straightforward tool to assess the efficacy of drug delivery systems in a lab environment.

## 1. Introduction

Molecular imprinting technology originally focused on the development of imprinted particles that could be packed into columns for affinity separation, exploiting the affinity and selectivity to extract a molecule of interest from complex matrices [1,2,3]. The concept was soon extended by using molecularly imprinted polymers (MIPs) as antibody or enzyme mimics [4,5]. One of the most interesting applications for MIPs is their incorporation into biomimetic sensing devices as they mimic the affinity a natural receptor has for its target but are superior in terms of their chemical, thermal and long term stability [6,7,8] and can be made by way of a straightforward and relatively low-cost synthesis process [9]. Although MIPs have been combined with optical [10,11], electrochemical [12,13,14,15], microgravimetric [16,17] and thermal transducers [18,19,20,21,22] in devices with great potential for e.g., diagnostic applications, the translation of these lab-based devices into commercially available diagnostic sensors is still challenging due to difficulties with reproducibility, integrated sampling and automated signal processing [23]. Therefore, this paper illustrates a new potential application for MIP-based sensing systems, i.e., the study of the elution kinetics of drug delivery systems.

Nanoporous matrices are macro-scale materials consisting of a large number of nano-scale pores [24] giving the material a high adsorption capacity with favorable mechanical properties over other types of nanomaterials [24]. As drug delivery systems, nanoporous materials are typically utilized to control drug release rate with a wide variety of compounds investigated for this purpose to date [25]. As with other types of nanostructured materials, the large amount of binding cavities can be used to store drugs for steady release [26]. The solubility of drugs that poorly dissolve in water can be regulated by carefully tuning the composition of the polymer and balancing the ratio of lipophilic and hydrophilic segments [27]. Over the past ten years, nanoporous metal-organic frameworks have emerged as an interesting class of nanomaterials due to their non-toxic nature and unusually large loadings of a wide variety of drugs [28]. This type of material can therefore be considered to be a suitable matrix for a drug delivery system. In addition, they can be synthesized without using organic solvents and have shown to release drugs in a gradual and sustaining manner which can be tuned by modifying the organic linkers within their binding cavities [29].

Drug elution kinetics from smart drug delivery systems are traditionally studied by measuring the concentration of the drug in the medium surrounding the drug carrier. The drug concentration in the elute is usually determined by classic laboratory devices/techniques including radioactive assays [29], fluorescent resonance energy transfer [30], liquid chromatography [31] and UV–Visible spectroscopy [32]. Although these methods are usually very sensitive and allow for a very accurate determination of the drug concentration and hence the elution profile, most of them require expensive readout devices, involve target labeling, or suffer from interference, limiting their performance in complex media. Therefore, selective, label-free techniques have been studied based on e.g., electrochemical [33] and microgravimetrical [34] readout methods. These platforms allow for a straightforward and relatively fast analysis of the eluted medium but still require ultrasensitive, expensive readout technology and conductive electrodes. In addition, data processing and interpretation can be tricky and require some experience.

Therefore, we introduce an elegant MIP-based platform for studying drug elution from a metal-organic framework in this article. Silver metalorganic frameworks were synthesized crosslinking AgNO_3_ with ethylene diamine in sodium-hydroxide solution leading to a sponge-like structure containing a wide distribution of nanometer sized cavities. These nanoporous matrixes were loaded with the model drug, acetylsalicylic acid (aspirin), one of the drugs most abundantly used for the treatment of pain, fever or inflammation that has also demonstrated to possess antithrombotic effects which positively affects patients at risk for heart failure [35]. The drug elution under physiological conditions was studied in the lab by applying aspirin-imprinted MIPs to aluminum electrodes and studying the propagation of a thermal wave through the chip in function of an increasing concentration of acetylsalicylic acid, a technique that was used successfully in previous work for the detection of dopamine in banana juice [21]. Next, the constructed dose-response curve from this experiment was used to determine the aspirin concentration in PBS solution containing drug eluted from the nanoporous matrix. The results demonstrated that the nanoporous matrix releases the drugs in a burst-like manner, releasing the drug in a constant fashion during the first two hours, after which the concentration in the media surrounding the sponge remains stable. These results were validated using UV–Visible spectroscopy, which shows a similar behavior and a similar concentration range, illustrating the potential of the platform for drug elution studies.

## 2. Materials and Methods

### 2.1. Chemicals

Acrylamide (AA), azobisisobutyronitrile (AIBN), polyvinylchloride (PVC) and silver nitrate (AgNO_3_) were obtained from Sigma-Aldrich, Zwijndrecht, The Netherlands. Methanol absolute and acetonitrile were bought at Biosolve B.V., Valkenswaard, The Netherlands. Acetone pure (purity > 99.9%), sodium hydroxide, sulfuric acid, ethanol, salicylic acid and phosphate buffered saline (PBS) tablets were acquired from VWR chemicals, Amsterdam, The Netherlands. Ethylenediamine (EDA), hydrazine hydrate and ethylene glycol dimethacrylate (EGDM) were procured from Merck Schuchardt OHG, Hohenbrunn, Germany. Polydimethysiloxane (PDMS) stamps were made with the Sylgard 184 elastomer kit from Mavom NV, Schelle, Belgium. Aluminum chips were purchased at Brico NV, Korbeek Lo, Belgium, and cut to the desired dimensions.

### 2.2. Instrumentation

Scanning electron microscopy (SEM) analysis of nanoporous matrices was performed with an SEM (Philips XL 30, M4I, Maastricht University, Maastricht, The Netherlands), at an operating voltage of 20 kV. Benchmarking with UV-Vis spectroscopy was done on a Shimadzu UV-3600 UV-VIS-NIR spectrophotometer (Shimadzu Europe, Duisburg Germany). Grinding and sieving of bulk MIPs were done using a Fritsch Planetary Micro Mill Pulverisette 7 premium line (VWR International, Amsterdam, The Netherlands) and a Fritsch Analysette (VWR International, Amsterdam, The Netherlands) with a 20 µm mesh, respectively.

The thermal detection platform used for studying elution has been described thoroughly in previous work [18,19,20,21,22]. Functionalized chips (MIP or NIP) were pressed mechanically with their backside onto a copper block serving as a heat provider. The temperature of the copper underneath the sample, *T*_1_, was monitored by a K-type thermocouple (TC Direct, Nederweert, The Netherlands). This information was fed into a temperature control unit that stringently controlled *T*_1_ by modifying the voltage over the power resistor (Farnell, Utrecht, The Netherlands) that heated the copper, using a software-based (Labview, National Instruments, Austin, TX, USA) proportional-integral-derivative (PID) controller (*P* = 10, *I* = 8, *D* = 0). The functionalized side of the chip faced a polyether ether ketone (PEEK) flow cell which was sealed with an O-ring to avoid leakage, defining a contact area of 28 mm^2^ and an inner volume of 110 µL. The flow cell was connected to a tubing system, allowing the administration of liquids in a controlled and automated fashion by means of a syringe pump. The temperature of the liquid inside the flow cell, *T*_2_, was measured by a second thermocouple, placed 1 mm above the chip. For each rebinding measurement the signal was stabilized in PBS at pH 7.4 which was used as to mimic physiological conditions. 

### 2.3. Synthesis of the Silver Nanoporous Matrix

Nanoporous matrixes were synthesized by mixing aqueous AgNO_3_ (5 mL, 0.4 M, 2 mmol) with aqueous NaOH (150 mL, 15 M, 2.25 mol). Crosslinking was initiated by the addition of aqueous EDA (1.5 mL, 99% *w/v*, 0.25 mmol) and aqueous hydrazine hydrate (0.2 mL, 80% *w/v*, 5.0 mmol) to the solution. The mixture was then purged with N_2_ and refluxed at 80 °C for 90 minutes with continuous stirring at 1500 rpm. After cooling the reaction flask to room temperature, silver nanosponges were isolated by vacuum filtration and air dried before being freeze dried for 6 hours. The resulting sponges had a loading efficiency of 73.4% ± 5.6% and a corresponding loading capacity of 65.6% ± 2.9%.

### 2.4. Molecular Imprinting Procedure

Pre-polymerization mixtures composed of aspirin—(0.090 g, 0.5 mmol), AA (0.213 g, 3 mmol)—and EGDM—(3.00 mL, 98% *w/v*, 15 mmol)—were dissolved in acetonitrile (5 mL). Polymerization was initiated by the addition of AIBN (0.025 g, 0.15 mmol) to the pre-polymerization mixture. The mixture was sonicated for 10 min, then deoxygenated by purging with N_2_ for 5 min. It was then heated to 60 °C during 24 hours while shielding the mixture from light to prevent the template from degrading. The resulting monolith was mechanically ground (700 rpm, 5 min, 10 mm balls) and sieved for 4 with a 20 µm mesh. The resulting powder was extracted for 96 hours at 105 °C, using a Soxhlet apparatus (VWR International, Amsterdam, The Netherlands) filled with a mixture of methanol and ethanoic acid (7:3 *v/v*) in order to remove the template, aspiring, from the MIP. Finally, the MIP particles were dried for three hours in an oven at 50 °C. Non-imprinted polymers (NIPs), serving as a reference, were synthesized in the same manner without the presence of a template. 

### 2.5. Chip Preparation

Polished aluminium plates were cut to obtain chips with the desired dimensions (10 × 10 mm^2^). To immobilize MIP particles onto the surface of the measurement chip, a 100 nm PVC adhesive layer (0.35 wt % PVC dissolved in tetrahydrofuran) was applied onto the chip by spin coating at 3000 rpm for 60 s with an acceleration of 1100 rpm/s. MIP and NIP particles were stamped into this layer using a PDMS substrate that was covered with a monolayer of polymer particles. The PVC layer was heated for 2 h at a temperature of 100 °C—chosen specifically to be significantly above its glass transition temperature (80 °C)—which allows beads to sink into the polymer layer. The samples were cooled down prior to thermal measurements and any unbound particles were washed off with distilled water.

### 2.6. Loading of Nanoporous Matrixes with Aspirin 

Aspirin was absorbed into the nanoporous matrixes by solvent evaporation. To this extent the nanosponge (0.100 g, 1.85 mmol), was incubated with aspirin (0.26 g, 1.85 mmol) in ethanol (6.2 mL). The mixture was shaken for 48 hours at 750 rpm. The solvent was removed under vacuum (30 °C, 300 mbar, 90 rpm) and dried at 65 °C for 3 h.

### 2.7. Drug Elution Analysis

Loaded nanoporous matrices (0.1 g) were incubated in 300 mL PBS (pH 7.4 at 37 °C) while gently stirring at 100 rpm to mimic physiological conditions. Over the course of two days, 3 mL aliquots of the PBS solution were taken at regular time intervals and the aspirin concentration was analyzed by both thermal wave transfer analysis (TWTA) and UV–Visible spectroscopy to create an elution profile.

## 3. Results and Discussion

### 3.1. Surface Characterization of Ag Nanosponges

**Figure 1:**
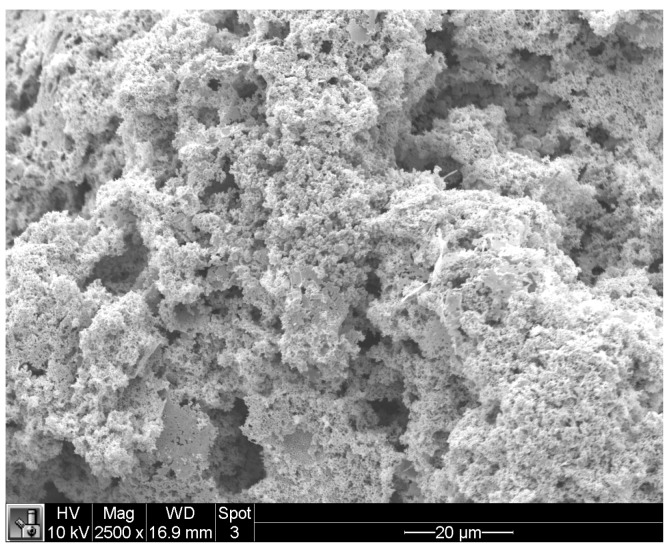
Surface analysis of an Ag-nanoporous matrix e using a scanning electron microscope at magnification 2500×.

The Ag nanosponges were analyzed using scanning electron microscopy (SEM). This analysis, shown in Figure 1, confirms that the metal-organic framework has a nanosponge structure containing a large set of nano-sized cavities, providing a large a surface area that can be loaded with drug molecules.

### 3.2. Quantification of Aspirin in PBS

**Figure 2:**
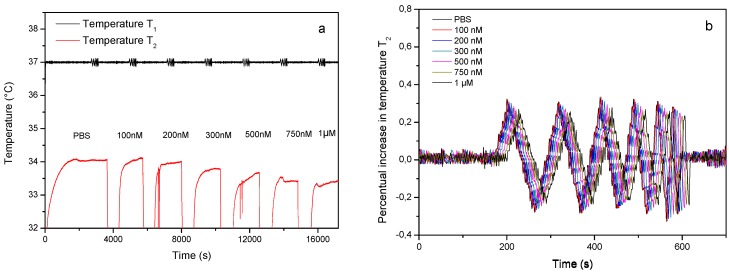

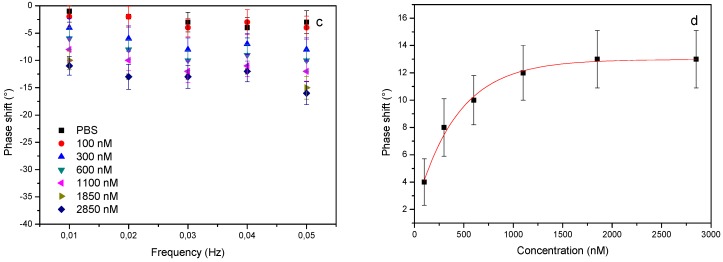
Aspirin rebinding analysis. The time-dependent temperature data (**a**) and thermal wave transport analysis spectrum (**b**) are shown in response to adding an increasing concentration of aspirin. The phase shift at an optimal resolution at frequency 0.03 Hz (**c**) and a dose-response curve (**d**) are constructed from these data and are plotted in function of the cummulative concetration present in the flow chamber.

To assess whether it was possible to accurately determine the concentration of drug eluted from the nanoporous matrix, a dose-response curve was constructed by exposing a MIP-coated chip to an increasing concentration of aspirin in PBS. The thermal analysis clearly indicates that exposing the MIP to aspirin in increasing concentrations results in a decrease of the liquid temperature inside the flow chamber (Figure 2a) and an increase in the phase shift observed in the transmitted wave (Figure 2b). The results in Figure 2b were used to construct a thermal Bode plot which shows the phase shift for each transmitted frequency in function of the cummulative concentration of aspirin present in the flow cell (Figure 2c). The error bars represent the maximum error observed on the measurement signal and was calculated by comparing a programmed temperature ramp with the actual temperature profile. Although the phase shift at every concentration is most pronounced at the highest input frequency, the resolution appears to be optimal at 0.03 Hz. These findings are in line with previously obtained results with dopamine MIPs in a similar setup [21]. The time-dependent TWTA data at 0.03 Hz were used to construct a dose-response curve (Figure 2d), which will be used to assess the concentration of aspirin that has eluted from the Ag-nanoporous matrix.

### 3.3. Selectivity Test

**Figure 3:**
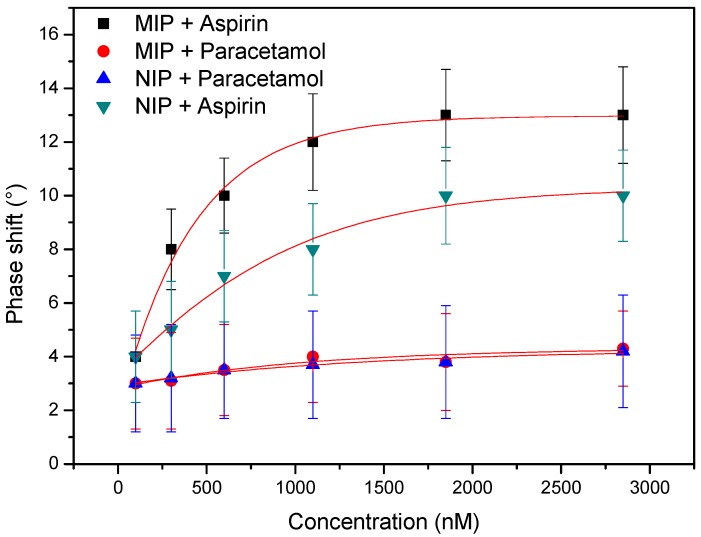
Molecularly imprinted polymer (MIP) selectivity test. The data show that exposing both the aspirin MIP and the Non-imprinted polymer (NIP) do not respond to an increasing concentration of the analogue molecule. However, the imprinting factor is limited as the difference between MIP and NIP is small.

In order to assess whether the aspirin recognition was selective and specific, the experiment summarized in the previous section was repeated for a NIP-coated electrode. In addition, both MIP and NIP were exposed to an increasing concentration of acetaminophen (paracetamol). The resulting dose-response curves and the corresponding fits are shown in Figure 3.

The results in Figure 3, illustrate that although the MIP is surprisingly selective in discriminating between paracetamol and aspirin, the imprinting factor is small. This conclusion is in line with previous findings with similar AA-based MIPs and can be explained by the fact that at a neutral pH, not all binding sites and functional groups on the target will be protonated (*pK*_a_ of aspirin is 3.49) [36]. Although previous work has indicated that the MIP would be more specific at acidic pH, the authors decided to continue measuring at a pH of 7.4 to simulate drug elution in physiological conditions. The results in Figure 2 indicate that the dose-response curve is highly usable and as the release pattern will be studied in PBS no interference from other molecules is to be expected. However, if the concept was extended to complex matrices in the future, the MIP synthesis route should be revised.

### 3.4. Thermal Analysis of Drug Elution

**Figure 4:**
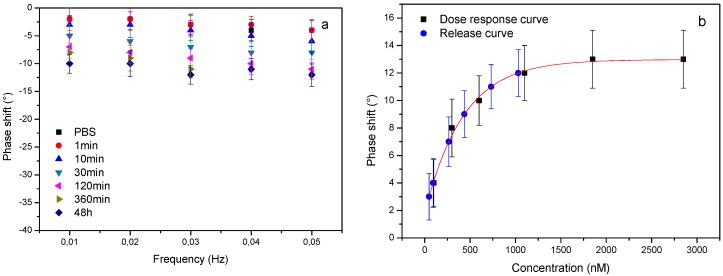
Drug elution analysis. The drug elution was studied using thermal wave transfer analysis (TWTA) and the resulting Bode plot shows a concentration-dependent phase shift indicating that the concentration of aspirin gradually increases with time (**a**). The dose-response curve was used to determine the concentration in the eluted solutions (**b**).

The elution of aspirin from the nanoporous matrixes was studied by incubating them in PBS and retrieving a sample from the surrounding medium after 1, 10, 30, 120 and 360 minutes and after 48 hours. The elutions were diluted 5000 times with PBS to fit the linear range of the sensor. MIP-coated electrodes were exposed to these diluted elutions and their response was summarized in a temperature Bode plot (Figure 4a). The resulting phase shifts at 0.03 Hz were used to construct an elution profile that was compared to the previously obtained dose response curve (Figure 4b) to determine the aspirin concentration in each of the eluted samples.

**Figure 5:**
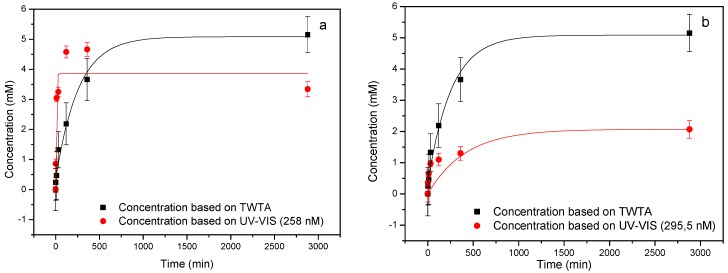
Validation of TWTA data by UV–Visible spectroscopy. The drug elution profile derived from the TWTA data in Figure 4 were compared to the drug elution profile obtained with UV–Visible and both show a initial burst of aspirin release in the first two hours in the milimolar regime (**a**). The decrease in aspirin concentration for the UV–Visible data shown in Figure 5a can be explained by conversion of aspirin into salicylic acid which is confirmed by analyzing absorbance at 295.5 nm (**b**).

The results obtained in Figure 4 were corrected for the dilution factor to create an elution profile. To benchmark the results obtained with our platform, the results were validated using a gold standard, relatively low-cost reference technique, i.e., UV–Visible spectroscopy/The elution profiles of both techniques demonstrate a similar behavior (Figure 5a): a characteristic exponential release pattern (fitted in Origin 8.0, Originlab, Northampton, MA, USA) which contains a sharp increase within the first two hours after which a stable plateau is reached that does not significantly change over the next two days. This indicates that the nanoporous matrix releases the drugs in a relatively quick burst which is suitable for some applications requiring immediate effect. However, to actually achieve sustained, prolonged delivery of drugs the nanoporous matrix should be functionalized with molecules that bind the drug and actually release it slowly over time.

When analyzing the elution profile obtained by UV–Visible spectroscopy, a small decrease in the concentration of aspirin can be observed over the course of two days, which leads to a distortion of the exponential fit. This can be explained by the fact that some of the acetylsalicylic acid will be converted into salicylic acid in PBS. This is confirmed by analyzing UV absorbance at 295.5 nm, which shows that salicylic acid is indeed present in the elution and its concentration will increase slightly over the course of two days (Figure 5b). The fact that this is not shown in the TWTA data is due to the fact that both compounds will bind to the MIP in a similar manner [36]. These data suggest that although the proposed sensor platform is not able to monitor the conversion of the drug to its metabolite, and is therefore less specific in this case, it is still a valuable alternative for the gold standard. Moreover, the application of UV–Visible spectroscopy for determining drug concentration in complex media can be troublesome due to the presence of other compounds that overlap the absorption spectrum of the drug under study [37]. The current platform on the other hand, has already proven to be capable of identifying small molecules in complex media such as blood plasma [38]. In addition, recent research has shown that the MIP-based thermal setup could be integrated into catheter-based commercial sensing devices which would enable to study drug release in vivo which is not possible with the current state-of-the-art techniques [39].

## 4. Conclusions

The data shown in this article illustrate the potential use of a MIP-based thermal detection platform, which has previously been used for diagnostic purposes, for analyzing the drug release kinetics of drug delivery matrices. A proof-of-principle was demonstrated by validating the results obtained with the biomimetic sensor using UV–Visible spectroscopy, demonstrating a similar profile in the same concentration range. The metalorganic framework synthesized in this work appears to release the model drug, aspirin, within the first two hours limiting its pharmaceutical use at this point. Therefore, future research should be aimed at functionalizing the framework to get to a more gradual release of the drug. In addition, its release profile will be studied in a more realistic setting to mimic sink conditions and potential degradation of the matrix to fully understand the pharmaceutical potential of diverse drug delivery systems. The proposed platform could be a very versatile tool for these release studies by providing valuable information on the performance of the drug release system. This information can in turn be used as a feedback loop to fine-tune the drug release framework’s synthesis procedure to acquire drug release matrices with a high potential for pharmaceutical application. In addition, loading and elution of other, potentially more relevant drugs should also be studied in more challenging, biological media. As the MIP platform is generic, it can be used to study a wide variety of targets in a wide variety of matrices, by changing the MIP receptors or optimizing their selectivity or performance in more challenging media and chemical conditions (pH, temperature, etc.).

## References

[1] Mosbach K., Mosbach R. (1966). Entrapment of enzymes and microorganisms in synthetic cross-linked polymers and their application in column techniques. Acta Chem. Scand..

[2] Sellergen B., Ekberg B., Mosbach K. (1985). Molecular imprinting of amino acid derivatives in macroporous polymers: Demonstration of substrate- and enantio-selectivity by chromatographic resolution of racemic mixtures of amino acid derivatives. J. Chromatogr..

[3] Schirhagl R., Hall E.W., Fuereder I., Zare R.N. (2012). Separation of bacteria with imprinted polymeric films. Analyst.

[4] Vlatakis G., Andersson L.I., Müller R., Mosbach K. (1993). Drug assay using antibody mimics made by molecular imprinting. Nature.

[5] Andersson L.I., Mosbach K. (1989). Molecular imprinting of the coenzyme-substrate analogue *N*-pyridoxyl-l-phenylalaninanilide. Makromol. Chem. Rapid Commun..

[6] Chianella I., Guerreiro A., Moczko E., Caygill J.S., Piletska E.V., De Vargas Sansalvador I.M.P., Whitcombe M.J., Piletsky S.A. (2013). Direct Replacement of Antibodies with Molecularly Imprinted Polymer Nanoparticles in ELISA—Development of a Novel Assay for Vancomycin. Anal. Chem..

[7] Haupt K., Mosbach K. (2000). Molecularly Imprinted Polymers and Their Use in Biomimetic Sensors. Chem. Rev..

[8] Ye L., Haupt K. (2004). Molecularly Imprinted Polymers as Antibody and Receptor mimics for Assays, Sensors and Drug Discovery. Anal. Bioanal. Chem..

[9] Whitcombe M.J., Kirsch N., Nicholls I.A. (2014). Molecular Imprinting Science and Technology: A Survey of the Literature for the Years 2004–2011. J. Mol. Recogn..

[10] Cennamo N., D’Agostino G., Pesavento M., Zeni L. (2014). High selectivity and sensitivity sensor based on MIP and SPR in tapered plastic optical fibers for the detection of l-nicotine. Sens. Actuators B Chem..

[11] Altintas Z., Gittens M., Guerreiro A., Thompson K., Walker J., Piletsky S., Tothill I.E. (2015). Detection of Waterborne Viruses Using High Affinity Molecularly Imprinted Polymers. Anal. Chem..

[12] Ramanaviciene A., Ramanavicius A. (2004). Molecularly Imprinted Polypyrrole-Based Synthetic Receptor for Direct Detection of Bovine Leukemia Virus Glycoproteins. Biosens. Bioelectron..

[13] Lakshimi D., Bossi A., Whitcombe M.J., Chianella I., Fowler S.A., Subrahmanyam S., Piletska E.V., Piletsky S.A. (2009). Electrochemical Sensor for Catechol and Dopamine Based on a Catalytic Molecularly Imprinted Polymer-Conducting Polymer Hybrid Recognition Element. Anal. Chem..

[14] Cai D., Ren L., Zhao H., Xu C., Zhang L., Ying Y., Wang H., Lan Y., Roberts M.F., Chuang J.H. (2010). A Molecular-Imprint Nanosensor for Ultrasensitive Detection of Proteins. Nat. Nanotechnol..

[15] Bajwa S.Z., Lieberzeit P.A. (2015). Recognition principle of Cu^2+^-imprinted polymers—Assessing interactions by combined spectroscopic and mass-sensitive measurements. Sens. Actuator B Chem..

[16] Ratautaite V., Plausinaitis D., Baleviciute I., Mikoliunaite L., Ramanaviciene A., Ramanavicius A. (2015). Characterization of Caffeine-Imprinted Polypyrrole by a Quartz Crystal Microbalance and Electrochemical Impedance Spectroscopy. Sens. Actuator B Chem..

[17] Hayden O., Dickert F.L. (2001). Selective Microorganism Detection with Cell Surface Imprinted Polymers. Adv. Mater..

[18] Wackers G., Vandenryt T., Cornelis P., Kellens E., Thoelen R., De Ceuninck W., Losada Pérez P., van Grinsven B., Peeters M., Wagner P. (2014). Array Formatting of the Heat-Transfer Method (HTM) for the Detection of Small Organic Molecules by Molecularly Imprinted Polymers. Sensors.

[19] Eersels K., van Grinsven B., Khorshid M., Somers V., Püttmann C., Stein C., Barth S., Diliën H., Bos G.M.J., Germeraad W.T.V. (2015). Heat-Transfer-Method-Based Cell Culture Quality Assay through Cell Detection by Surface Imprinted Polymers. Langmuir.

[20] Van Grinsven B., Eersels K., Akkermans O., Ellermann S., Kordek A., Peeters M., Deschaume O., Bartic C., Diliën H., Steen Redeker E. (2016). Label-Free Detection of Escherichia Coli Based on Thermal Transport Through Surface Imprinted Polymers. ACS Sens..

[21] Peeters M.M., van Grinsven B., Foster C.W., Cleij T.J., Banks C.E. (2016). Introducing Thermal Wave Transport Analysis (TWTA): A Thermal Technique for Dopamine Detection by Screen-Printed Electrodes Functionalized with Molecularly Imprinted Polymer (MIP) Particles. Molecules.

[22] Steen Redeker E., Eersels K., Akkermans O., Royakkers J., Dyson D., Nurekeyeva K., Ferrando B., Cornelis P., Peeters M., Wagner P. (2017). Biomimetic Bacterial Identification Platform Based on Thermal Wave Transport Analysis (TWTA) through Surface-Imprinted Polymers. ACS Inf. Dis..

[23] Selvolini G., Marrazza G. (2017). MIP-Based Sensors: Promising New Tools for Cancer Biomarker Determination. Sensors.

[24] Lu G.Q., Zhao X.S., Lu G.Q., Zhao X.S. (2004). Nanoporous Materials—An Overview. Nanoporous Materials Science and Engineering.

[25] Fukumori Y., Takada K., Takeuchi H., Kumar C.S.S.R. (2007). Nanoporous and Nanosize Materials for Drug Delivery Systems. Nanomaterials for Medical Diagnosis and Therapy.

[26] Subramanian S., Singireddy A., Krishnamoorthy K., Rajappan M. (2012). Nanosponges: A Novel Class of Drug Delivery System—Review. J. Pharm. Pharm. Sci..

[27] Cavalli R., Trotta F., Tumiatti W. (2007). Cyclodextrin-based Nanosponges for Drug Delivery. J. Incl. Phenom. Macrocycl. Chem..

[28] Horcajada P., Serre C., Vallet-Regi M., Sebban M., Taulelle F., Férey G. (2006). Metal–Organic Frameworks as Efficient Materials for Drug Delivery. Angew. Chem. Int. Ed..

[29] Horcajada P., Chalati T., Serre C., Gillet B., Sebrie C., Baati T., Eubank J.F., Hertaux D., Kreuz C., Chang J.S. (2010). Porous metal–organic-framework nanoscale carriers as a potential platform for drug delivery and imaging. Nat. Mater..

[30] Bagalkot V., Zhang L., Levy-Nissenbaum E., Jon S., Kantoff P.W., Langer R., Farokhzad O.C. (2007). Quantum Dot-Aptamer Conjugates for Synchronous Cancer Imaging, Therapy, and Sensing of Drug Delivery Based on Bi-Fluorescence Resonance Energy Transfer. Nano Lett..

[31] Wang Q., Ma D., Higgins P.J. (2006). Analytical method selection for drug product dissolution testing. Dissolut. Technol..

[32] Kamberi M., Tran T.N. (2012). UV-visible spectroscopy as an alternative to liquid chromatography for determination of everolimus in surfactant-containing dissolution media: a useful approach based on solid-phase extraction. J. Pharm. Biomed. Anal..

[33] Lee R., Jo D.H., Chung S.J., Na H.K., Kim J.H., Lee T.G. (2016). Real-time and label-free monitoring of nanoparticle cellular uptake using capacitance-based assays. Sci. Rep..

[34] Karlsson J., Atefyekta S., Andersson M. (2015). Controlling drug delivery kinetics from mesoporous titania thin films by pore size and surface energy. Int. J. Nanomed..

[35] Miner J., Hoffhines A. (2007). The Discovery of Aspirin’s Antithrombotic Effects. Tex. Heart Inst. J..

[36] Meischl F., Schemeth D., Harder M., Köpfle N., Tessadri R., Rainer M. (2016). Synthesis and evaluation of a novel molecularly imprinted polymer for the selective isolation of acetylsalicylic acid from aqueous solutions. J. Enivorn. Chem..

[37] Narayanan S., Orton S., Leparc G.F., Garcia-Rubio L.H. (1999). Ultraviolet and visible light spectrophotometric approach to blood typing: Objective analysis by agglutination index. Transfusion.

[38] Peeters M., Troost F.J., van Grinsven B., Horemans F., Alenus J., Murib M.S., Keszthelyi D., Ethirajan A., Thoelen R., Cleij T.J. (2012). MIP-based biomimetic sensor for the electronic detection of serotonin in human blood plasma. Sens. Actuator B Chem..

[39] Diliën H., Peeters M., Royakkers J., Harings J., Cornelis P., Wagner P., Steen Redeker E., Banks C.E., Eersels K., van Grinsven B. (2017). Label-Free Detection of Small Organic Molecules by Molecularly Imprinted Polymer Functionalized Thermocouples: Toward In Vivo Applications. ACS Sens..

